# A Novel Mouse Model of the Glucocorticoid Withdrawal Syndrome

**DOI:** 10.1210/jendso/bvaf116

**Published:** 2025-07-13

**Authors:** Christen N Snyder, Lana L Haddad, Oliver Rubin, Chih-Lin Chang, Joanna L Spencer-Segal

**Affiliations:** Neuroscience Graduate Program, University of Michigan, Ann Arbor, MI 48109, USA; Michigan Neuroscience Institute, University of Michigan, Ann Arbor, MI 48109, USA; College of Literature, Science, and the Arts, University of Michigan, Ann Arbor, MI 48109, USA; Michigan Neuroscience Institute, University of Michigan, Ann Arbor, MI 48109, USA; Michigan Neuroscience Institute, University of Michigan, Ann Arbor, MI 48109, USA; Michigan Neuroscience Institute, University of Michigan, Ann Arbor, MI 48109, USA; Division of Metabolism, Endocrinology, and Diabetes, Department of Internal Medicine, University of Michigan Medical School, Ann Arbor, MI 48109, USA

**Keywords:** glucocorticoid withdrawal syndrome, Cushing's syndrome, Cushing's disease, pain, social interaction

## Abstract

Glucocorticoid withdrawal syndrome (GWS) is increasingly recognized as a major barrier to discontinuing chronic glucocorticoid treatment. Despite high rates of adverse effects, 1% to 3% of the population continues long-term glucocorticoid use, in part due to this poorly understood syndrome. Prominent manifestations of GWS include generalized pain, asthenia, and decreased behavioral motivation, which are only reversed by resuming or increasing glucocorticoid use. Here, we present the first mouse model of GWS designed to investigate its underlying mechanisms, which are so far unknown. Male and female mice were administered chronic high-dose prednisolone via drinking water, followed by withdrawal to low levels. During withdrawal, male and female mice showed an increase in nociceptive behavior in the von Frey test. Female mice also showed decreased interaction with a novel conspecific, despite no change in classic affective behaviors, suggesting a sex-specific decrease in social motivation. We assessed brain glucocorticoid receptor (GR) expression via immunohistochemistry in brain regions involved in pain sensitivity and motivated behavior and found decreased optical density of GR immunoreactivity during chronic prednisolone treatment, specifically in the anterior cingulate cortex. In conclusion, we found that mice exhibit behavioral changes during glucocorticoid withdrawal that mimic the human GWS. This model can therefore be used to study the neural mechanisms of this poorly understood syndrome, including the role of region-specific changes in GR expression.

Since the Nobel Prize was awarded in 1950 for the discovery of cortisone and its use in treating rheumatoid arthritis and other diseases, glucocorticoids have become one of the most widely prescribed drug classes worldwide. While their potent immunosuppressive and anti-inflammatory effects revolutionized the treatment of autoimmune and inflammatory diseases, the adverse consequences of long-term glucocorticoid excess quickly became apparent, limiting their suitability for extended use [1-3]. Despite early recognition of these toxicities and the growing availability of steroid-sparing alternatives, an estimated 1% to 3% of the population still takes long-term glucocorticoids, contributing to substantial excess morbidity and mortality [4-6].

A major, yet often under-recognized, barrier to glucocorticoid discontinuation is the glucocorticoid withdrawal syndrome (GWS), a difficult-to-manage condition that remains poorly understood and lacks effective treatment. GWS can arise after the withdrawal of supraphysiological levels of glucocorticoids, whether endogenous or exogenous (eg, cortisol excess due to pituitary, adrenal, or other neuroendocrine tumors, or chronic glucocorticoid treatment). Its most prominent symptoms include generalized pain (myalgias and arthralgias) and fatigue with reduced motivation [7, 8]. Importantly, these symptoms often occur independently of autoimmune or inflammatory disease reactivation and can persist in the absence of hypothalamic-pituitary-adrenal (HPA) axis suppression [9-13]. This syndrome appears to depend on glucocorticoid receptor (GR) signaling as it can be pharmacologically mimicked by GR antagonism (eg, with mifepristone) and alleviated by glucocorticoid reinstatement [12, 14]. Despite increasing clinical recognition, the biological mechanisms of GWS remain completely unknown; however, the prominence of behavioral symptoms and generalized GWS-related pain suggest a central nervous system basis [8]. To address this gap, we aimed to develop an animal model of the GWS suitable for investigating its neural mechanisms.

The mesolimbic dopamine pathway, including the ventral tegmental area (VTA) and its projection areas in the striatum, prefrontal cortex, and periaqueductal gray (PAG), could play a central role in the GWS due to its glucocorticoid sensitivity and its role in pain and motivated behavior [15, 16]. GRs are expressed in approximately 50% of dopamine neurons in the VTA, and glucocorticoids have been shown to acutely increase VTA neuron activity [17]. Furthermore, GR signaling in dopaminoceptive neurons in the ventral striatum are necessary for the regulation of motivated behavior [18]. Indeed, in laboratory rodents, acute glucocorticoids regulate both pain sensitivity and behavioral motivation. For example, administration of dexamethasone 1 hour after sciatic nerve injury decreased mechanical allodynia in rats [19]. Glucocorticoids also acutely stimulate dopamine-dependent motivation for rewards in both animals and humans; however, these acute effects of glucocorticoids on motivated behavior are not sustained chronically [20-22].

In states of chronic glucocorticoid excess, cellular adaptations such as GR downregulation are known to occur [23-25]. The withdrawal syndrome may result from the combination of adaptive limitations to GR activity and a sudden drop in glucocorticoid availability, resulting in symptoms resembling absolute glucocorticoid deficiency. Prior studies suggest that glucocorticoid-induced GR downregulation does occur in the brain, although the variability in findings suggests that this phenomenon differs across exposure paradigms and brain regions [23, 26-28]. GR downregulation in the VTA and its projection areas during chronic glucocorticoid treatment could underlie changes in pain sensitivity and motivated behavior in the GWS.

The primary goal of this study was to develop the first animal model of GWS and to use it to begin investigating the neural mechanisms underlying the syndrome. To this end, we administered prednisolone in the drinking water for 8 weeks to male and female mice followed by a withdrawal phase, during which we assessed mechanical pain sensitivity and innate affective and motivated behavior. Finding that primary behavioral manifestations of GWS were recapitulated in the mice, we used this model to test the hypothesis that chronic prednisolone exposure leads to changes in GR expression in key brain regions implicated in these behaviors.

## Methods

### Animal Husbandry

Male and female C57Bl/6J mice at 4 to 6 months of age were obtained from the Jackson Laboratory. Two same-sex cohorts of 30 mice were divided equally into 3 experimental groups. Mice were group housed (5/cage) and maintained on a 14 hours/10 hours light/dark cycle where the lights came on at 06:00. Food and water were provided ad libitum throughout the duration of the study. All experimental protocols were approved by the University of Michigan Institutional Animal Care and Use Committee and were in accordance with the National Institutes of Health Guide for the Care and Use of Laboratory Animals.

### Glucocorticoid Treatment

**Figure 1.**
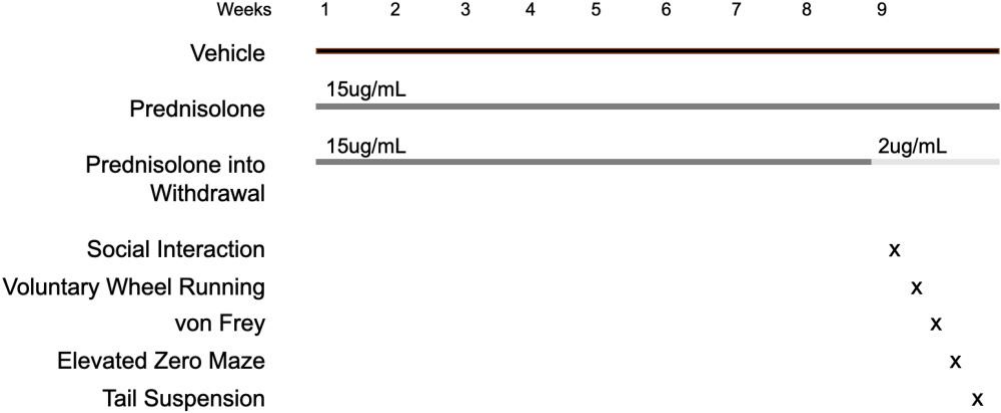
Glucocorticoid withdrawal paradigm and behavioral test battery. Mice were treated with prednisolone (via drinking water) or vehicle for 8 weeks, followed by 10 days of withdrawal or continued treatment during which behavioral testing occurred. Starting on withdrawal day 3, mice underwent 1 behavioral test per day. The test battery included the social interaction test, voluntary wheel running, von Frey, elevated zero maze, and tail suspension tests.

Mice were divided into 3 experimental groups defined by the drinking water they received. The control group received standard water for the duration of the study; the treatment group received 15 μg/mL of hemisuccinate-bound prednisolone (Sigma Aldrich, P4153) in the drinking water for the duration of the study, and the withdrawal group received 15 μg/mL of prednisolone for 8 weeks followed by 1 week of withdrawal to 2 μg/mL of prednisolone (Fig. 1).

### Animal Behavior

Mice were habituated to the testing room for 1 hour prior to behavioral testing. Arenas were cleaned with 50% ethanol in between animals. Mouse behavior was captured by top-down cameras (Basler acA1920-40umMED) unless otherwise noted and the positions of nose, tail, and center of mass were tracked using Ethovision XT software (Noldus Inc).

#### Social interaction

Mice were tested in a modified social interaction test on day 3 of withdrawal during the active (dark) phase to investigate preference for social contact with a novel conspecific [29]. Animals were placed in the center of a square arena (30 × 30 × 30 cm) under dim lighting conditions (55 lux) (Noldus Phenotyper). Two identical black mesh cups (8 cm diameter, 9 cm tall) were placed in opposite corners of the arena. The interaction zone for each cup was defined as a circular area 1 cm larger than the mesh cup. Animals were allowed to fully explore the arena with the empty mesh cups for 150 seconds (habituation session). Immediately after, the test mouse was removed from the Phenotyper while an age- and sex-matched novel conspecific mouse was placed under one of the mesh cups. The test mouse was then placed back into the arena and behavior recorded for an additional 150 seconds (testing session). Ethovision XT (Noldus Inc) was used to extract behavioral endpoints of total locomotion (cm), time spent in each interaction zone (s), and entries to the interaction zones. The social interaction ratio was calculated as the amount of time spent in the interaction zone with the conspecific divided by the total amount of time spent in either interaction zone during the testing session.

#### Voluntary wheel running

The Noldus Phenotyper cages were equipped with a freely accessible running wheel (diameter: 15 cm, width: 7 cm; PhenoWheel, Noldus Inc). Mice were placed within the cage and allowed to explore for 30 minutes while behavior was recorded. Behavioral endpoints measured included the number of wheel rotations, time spent in the wheel zone, and time spent moving in the wheel zone. In Ethovision, mobility was defined as a change of more than 2% in the mouse's detected area between consecutive frames.

#### Pain sensitivity

The ascending stimulus method of the von Frey test was used to evaluate mechanical allodynia [30, 31]. Mice were individually placed in small cages with mesh bottoms and allowed to acclimate for 90 minutes. Calibrated monofilaments (Bioseb Lab Instruments, BIO-VF-M) with increasing force (ranging from 0.008 to 4 g) were applied to the plantar surface of the hind paw until a withdrawal response was elicited, or for a maximum of 5 seconds. Each monofilament was tested 5 times per hind paw with between 3 and 5 monofilaments being used per mouse (∼20 monofilament presentations). A response was defined as a lifting, shaking, or licking of the paw upon stimulation or stimulation removal. If no response occurred within 5 seconds on 4 or more applications, the next strongest force was used. The withdrawal threshold was determined using a response rate of 40% or more (2 or more applications eliciting a response). The withdrawal thresholds for each paw were then averaged.

#### Elevated zero maze

On day 6 of withdrawal, mice were tested in the elevated zero maze (EZM), an elevated ring-shaped platform (inner diameter: 50 cm, outer diameter: 62 cm) consisting of 4 alternating open and closed areas (San Diego Instruments). The maze was 52 cm above the ground, with opaque walls in the closed area measuring 15 cm tall. Each mouse was placed in the closed area, facing the border to the open area, and allowed to explore for 10 minutes under bright lighting conditions (∼200 lux). Measured behavioral endpoints included total distance traveled (cm), distance traveled in the open areas (cm), number of entries to the open areas, and time spent in the open areas(s).

#### Tail suspension test

Medical grade tape was used to secure the mouse to a horizontal metal rod by its tail 20 cm above the table surface. A small cardboard tube was placed over the base of the tail to prevent the mice from tail climbing. A camera (Canon Vixia HF M31) positioned at a 90-degree angle toward the mouse was used to record behavior for 6 minutes. Immobility was defined as a change in <2% of the area detected as the animal per frame using mobility states in Ethovision XT (Noldus Inc).

### Blood Collection

The mice were placed beneath an infrared lamp 90 seconds prior to blood collection to cause venous dilation. Mice were then placed in a tail vein restrainer (Braintree Scientific, Cambridge, MA) and the tail vein was identified. The area was cleaned with a sterile alcohol prep pad and quickly incised with a razor blade. Microvette collection tubes with EDTA additive (Sarstedt Inc, 16.444.100) were used to collect blood for 60 seconds after the incision. Pressure was then applied to encourage hemostasis and petroleum jelly (Covidien, 8884433200) was placed over the incision. The mice were then returned to their home cage.

### Euthanasia and Tissue Collection

The mice received an intraperitoneal injection of a lethal dose of sodium pentobarbital (150 mg/kg). Once reflexes were absent, the chest cavity was opened, and an insulin syringe was used to collect cardiac blood over the course of 1 minute followed by an intracardiac perfusion with 20 mL of phosphate-buffered saline (PBS) and 20 mL of 4% paraformaldehyde (PFA) in PBS. Brains were retrieved and fixed in 4% PFA for 48-hours before sinking in ascending concentrations of sucrose (10%, 20%, and 30%). Brains were then flash frozen on dry ice prior to being placed in −80 °C until further use.

### Hormone Assays

All blood samples were centrifuged at 1500*g* (Fisher Scientific, 13-100-675) for 10 minutes at 4 °C. The supernatant was collected and frozen at −80 °C until further use. The Corticosterone DetectX Enzyme Immunoassay Kit (Arbor Assays, K014, RRID: AB_2877626) was used to measure corticosterone levels following manufacturer's instructions. The cross reactivity of this kit with prednisolone is minimal at 0.177% (Arbor Assays). Absorbance was read at 450 nm using a BioTek microplate reader (Agilent).

### Histology

Whole brains were sectioned in 30-µm slices using a sliding microtome (Leica SM2010R) and tissue was stored at −20 °C in cryoprotectant (30% sucrose and 50% ethylene glycol in PBS) to prevent freezing until use. Sections were washed in Tris-buffered saline (TBS) and 0.5% bovine serum albumin (BSA) for 30 minutes each, then incubated overnight at room temperature in 1:1000 rabbit anti-GR primary antibody (Cell Signaling Technology Cat# 3660, RRID: AB_11179215). The next day, tissue was rinsed for 45 minutes in TBS before being incubated in goat anti-rabbit IgG antibody biotinylated secondary (Vector Laboratories Cat# BA-1000, RRID: AB_2313606) for 30 minutes at room temperature. Sections were then washed in TBS for 45 minutes prior to being incubated for 30 minutes in avidin-biotin-peroxidase complex (ABC) solution (Vector Laboratories Cat# PK-6100, RRID: AB_2336819). After another 40 minutes wash with TBS, sections were incubated in 3,3′-diaminobenzidine (DAB) solution with DAB peroxidase substrate (Millipore Sigma, D12384), TBS, and 30% hydrogen peroxide (Millipore Sigma, 21673) for 8 minutes. TBS and 0.1M PBS were used to wash sections for 6 minutes each. Sections were then mounted on glass microscope slides (Fisher Scientific, 12-550-15) in 0.1M PBS and placed in the desiccator for 25 minutes. Sections were further dehydrated through an ascending alcohol series (75%, 80%, 90%, and 100%) finishing in xylenes. Permount (Fisher Scientific, SP15-100) mounting media was used to coverslip the slides.

### Immunohistochemical Analysis

Slides were imaged on an Olympus BH2 Fluorescent Microscope. Bright-field images were acquired with 4× and 10× objectives, maintaining consistent light and acquisition settings across all samples. Images were captured with an Olympus DP23M monochrome camera and StereoInvestigator software (MBF Biosciences).

Coronal sections were selected based on brain regions hypothesized to be involved in glucocorticoid withdrawal, guided by the Allen Brain Reference Atlas (Mouse, p56, Coronal; ABA) [32]. Four regions were analyzed: the anterior cingulate cortex (ABA image 60), nucleus accumbens (ABA image 45), ventral tegmental area (ABA image 82), and periaqueductal gray (ABA image 86). For each animal, a single section per brain with optimal morphology and consistent labeling was chosen for analysis. Some animals were excluded due to tissue quality issues or section loss.

Images were converted to 8-bit grayscale and further processed using NIH ImageJ. Background correction was performed using rolling ball method, which preserves low-intensity fine details while correcting for uneven illumination. The mean gray value within a selected region of interest was measured to determine the optical density (OD) of the sample. OD values were then normalized to the control. To obtain cell counts, the *analyze particles* feature was used, and the number of particles detected was divided by the total area of the region of interest.

### Statistical Analysis

Statistical analyses were conducted to assess the effects of sex and treatment on physiological, behavioral, and histological measures. Due to the significant weight difference between sexes, male and female weights were analyzed separately. For each sex, weights were normalized to baseline prior to analysis. Effects of prednisolone treatment on weight were assessed using a mixed-effects model for the first 8 weeks, followed by a 2-way repeated measures ANOVA for the withdrawal period (weeks 8-9). All post hoc comparisons were performed using Tukey's multiple comparisons test.

Insulin levels showed no sex differences, so data were pooled and analyzed using a one-way ANOVA with appropriate post hoc tests. In contrast, due to the known sex differences in baseline corticosterone levels, the corticosterone data were analyzed separately for males and females using 2-way repeated measures ANOVA. Fisher's least significant difference (LSD) test with manual Bonferroni correction was applied for post hoc comparisons.

Behavioral data were analyzed using a 2-way ANOVA (sex × treatment) to assess potential sex differences. Within sex, post hoc comparisons using Tukey's multiple comparisons test were performed to clarify the treatment effect. Additionally, the social interaction ratios were compared to the theoretical chance level using 1-sample *t* tests. Only the final 4 minutes of the tail suspension test were analyzed.

GR labeling across brain regions was analyzed using a one-way ANOVA to assess treatment effects. Sex was not included as a factor in the analysis due to tissue loss and compromised quality in some samples, which limited the representation of males or females in certain groups.

All analyses were performed in GraphPad Prism 9 (v10.4.0), with significance set at *P* < .05. Graphs display individual data points along with the group mean and standard error of the mean (SEM).

## Results

The primary goal of this study was to create the first mouse model of withdrawal from chronic glucocorticoid excess and determine whether it recapitulates features of the human GWS. To do so, prednisolone, the active metabolite of prednisone, was administered in the drinking water for 8 weeks as a noninvasive method for chronic glucocorticoid exposure. Prednisone and prednisolone are intermediate-acting synthetic glucocorticoids commonly used to treat patients with autoimmune and inflammatory disorders. Prednisolone is roughly 5 times more potent than the endogenous cortisol (humans) or corticosterone (rodents). The hemisuccinate-bound prednisolone complex was used to enhance solubility in the aqueous vehicle. Mice were treated continuously with high-dose (15 μg/mL) prednisolone or vehicle (standard water) or treated with high-dose prednisolone for 8 weeks then withdrawn to a low dose (2 μg/mL) during week 9 (Fig. 1). This low dose of prednisolone during withdrawal was used to prevent frank adrenal insufficiency, which can occur with prolonged suppression of the HPA axis. Behavioral testing began on day 3 of withdrawal.

### Chronic Prednisolone Treatment Results in Weight Loss and Insulin Resistance

**Figure 2.**
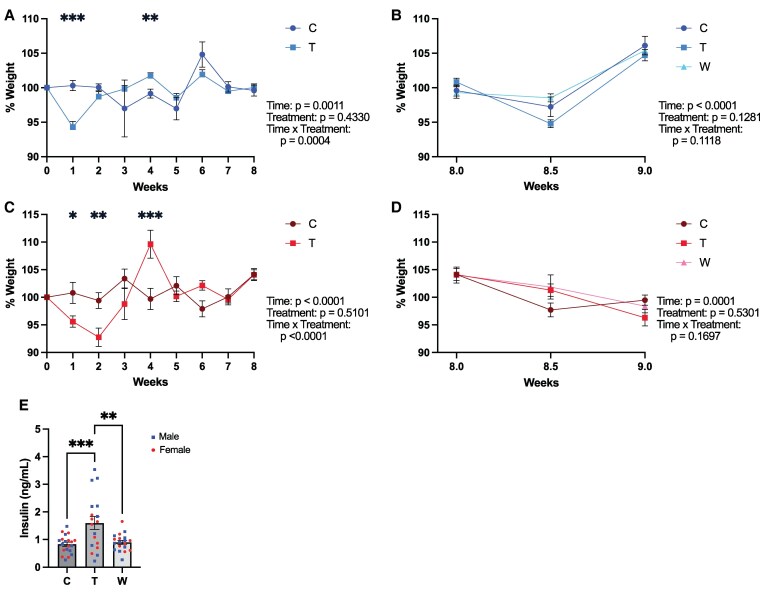
Glucocorticoid treatment alters body weight and insulin levels in male and female mice. A and B, Body weight changes in male mice during 8 weeks of prednisolone or vehicle treatment (A) and during continued prednisolone/vehicle treatment or withdrawal (B). C and D, Body weight changes in female mice during prednisolone or vehicle treatment (C) and during continued prednisolone/vehicle treatment or withdrawal (D). E, Serum insulin levels increased significantly during prednisolone treatment and decreased during withdrawal. Error bars represent standard error of the mean (SEM). *P < .05, **P < .005, ***P < .0005 in post hoc tests. Abbreviations: C, Control; T, Treatment; W, Withdrawal.

Chronic glucocorticoid treatment in humans often leads to metabolic changes, such as weight gain, decreased lean mass with increased fat mass, and insulin resistance which typically reverse during withdrawal [8, 33-35]. In rodents, the impact of glucocorticoids on weight is more variable. Previous literature has demonstrated that high doses of corticosterone can cause weight gain and hyperinsulinemia in rodents [36, 37]; however, treatment with dexamethasone has also been associated with weight loss and altered body composition (ie, increased fat mass and decreased lean mass) [38-40]. To assess the effects of chronic prednisolone treatment and withdrawal on the metabolic phenotype, we weighed mice weekly and measured insulin levels from blood collected at the time of perfusion (Fig. 2).

Both male and female mice initially lost weight during prednisolone treatment, followed by weight regain. This pattern was reflected in a significant interaction between time and treatment condition (males: F[8, 240] = 3.751, *P* = .0004; females: F[8, 239] = 4.610, *P* < .0001). This interaction indicates that the effect of chronic prednisolone on weight depended on the time since treatment initiation. Post hoc testing revealed that, in males, compared to controls, the treatment group had significantly lower weights at week 1 (*P* < .0001) and higher weights at week 4 (*P* = .0040). Similarly, in females, the treatment group had significantly lower weights than the controls at weeks 1 (*P* = .0288) and 2 (*P* = .0058) but higher at week 4 (*P* < .0001). There was also a main effect of time on body weight (males: F[3.030, 90.91] = 5.762, *P* = .0011; females: F[8, 239] = 4.963, *P* < .0001). There was no main effect of prednisolone treatment alone (males: F[1, 240] = 0.6168, *P* = .4330; females: F[1, 239] = 0.4352, *P* = .5101).

During the week of glucocorticoid withdrawal and behavioral testing, body weight changes differed between the sexes where males regardless of treatment group showed significant weight gain (main effect of time, F[1.725, 43.13] = 56.56, *P* < .0001) and females showed significant weight loss (main effect of time, F[1.612, 38.69] = 13.31, *P* = .0001). There was no significant effect of treatment during the withdrawal period (males: F[2, 25] = 2.234, *P* = .1281, females: F[2, 24] = 0.6518, *P* = .5301). Similarly, there was no interaction between treatment and weight change during withdrawal (males: F[4, 50] = 1.981, *P* = .1118, females: F[4, 48] = 1.682, *P* = .1697). Overall, these findings suggest that chronic prednisolone treatment temporarily alters weight trajectories in both male and female mice. Weight changes during behavioral testing, however, are sex-specific and independent of treatment.

There was no effect of sex on insulin levels at the time of perfusion and the sexes were pooled for analysis. A one-way ANOVA revealed a significant main effect of treatment on insulin levels (F[2, 54] = 8.866, *P* = .0005). Post hoc comparisons showed that mice receiving prednisolone had significantly higher insulin levels than both control mice (*P* = .0009) and mice in the withdrawal group (*P* = .003). These results suggest that chronic prednisolone treatment induces insulin resistance, mimicking the metabolic effects of glucocorticoid excess in humans. This effect resolved within 1 week of withdrawal.

### Chronic Prednisolone Treatment Inhibits the HPA Axis

**Figure 3.**
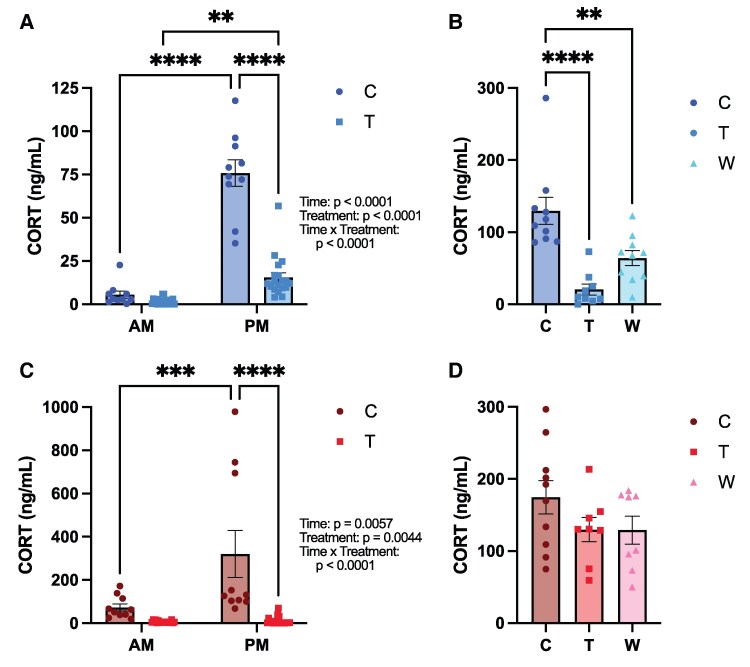
Prednisolone treatment reduces circulating corticosterone levels in male and female mice. A, Morning and evening corticosterone levels in male mice 4 weeks into treatment. B, Corticosterone levels at the time of perfusion in male mice. C, Morning and evening corticosterone levels in female mice under the same treatment conditions. D, Corticosterone levels at the time of perfusion in female mice. Statistical results listed on the graphs are from 2-way ANOVA with sex and treatment as independent variables. Error bars represent SEM. *P < .05, **P < .005, ***P < .0005, ****P < .0001 in post hoc tests. Abbreviations: C, Control; T, Treatment; W, Withdrawal.

Glucocorticoids suppress the HPA axis via feedback inhibition. The goal of this model was to administer chronic supraphysiologic doses of glucocorticoid, which is expected to induce HPA axis suppression, as occurs in humans with prolonged endogenous or exogenous glucocorticoid exposure [41]. To assess the impact of prednisolone treatment on endogenous HPA axis activity, we measured circulating corticosterone levels in male and female mice during week 4 of treatment, at both morning and evening time points (Fig. 3A and 3C). As expected, based on previous literature, female mice exhibited higher overall corticosterone levels than males. Corticosterone data from each sex were analyzed separately.

In both sexes, the control group showed the expected diurnal rhythm of corticosterone, and the treatment group showed evidence of HPA axis suppression. In males, a 2-way ANOVA showed a main effect of time of day (F[1, 27] = 159.6, *P* < .0001), treatment (F [1, 27] = 82.56, *P* < .0001), and an interaction between time of day and treatment (F[1, 27] = 73.29, *P* < .0001). HPA axis suppression was demonstrated as the evening corticosterone levels were significantly lower in the treatment group vs controls (*P* < .0001). Despite that, male mice in the prednisolone group retained some diurnal variation, with the morning and evening corticosterone levels remaining significantly different within both groups (control: *P* < .0004, treatment: *P* = .0072).

In females, a 2-way ANOVA also revealed a main effect of time of day (F[1, 26] = 9.718, *P* = .0044), treatment (F[1, 26] = 21.69, *P* < .0001), as well as an interaction between time and treatment (F[1, 26] = 9.071, *P* = .0057). As in the males, the evening corticosterone levels were significantly higher in controls than in treated mice (*P* < .0001). Females exhibited more robust HPA axis suppression with prednisolone than males, such that only control mice showed a significant evening rise in corticosterone (*P* = .0028), whereas prednisolone-treated females did not (*P* > .9999). Together, these data confirm that chronic prednisolone effectively suppresses endogenous corticosterone production, with more profound suppression in females than in males.

To investigate the persistence of HPA axis suppression during continued vehicle/prednisolone treatment or withdrawal, corticosterone levels were also measured from cardiac blood collected at the time of perfusion (Fig. 3B and 3D). A one-way ANOVA revealed a significant effect of treatment on corticosterone levels in male mice (F[2, 26] = 0.9334, *P* < .0001). Post hoc comparisons showed that both treated (*P* < .0001) and withdrawal (*P* = .0046) groups had significantly lower corticosterone levels than controls. The male withdrawal group showed a trend toward HPA axis recovery; however, the comparison between treatment withdrawal groups did not reach significance (*P* = .0815). In contrast, in females, treatment had no significant effect on circulating corticosterone levels at the time of perfusion (F[2, 23] = 1.701, *P* = .2046).

### Prednisolone Withdrawal Alters Social Interaction in Female Mice

**Figure 4.**
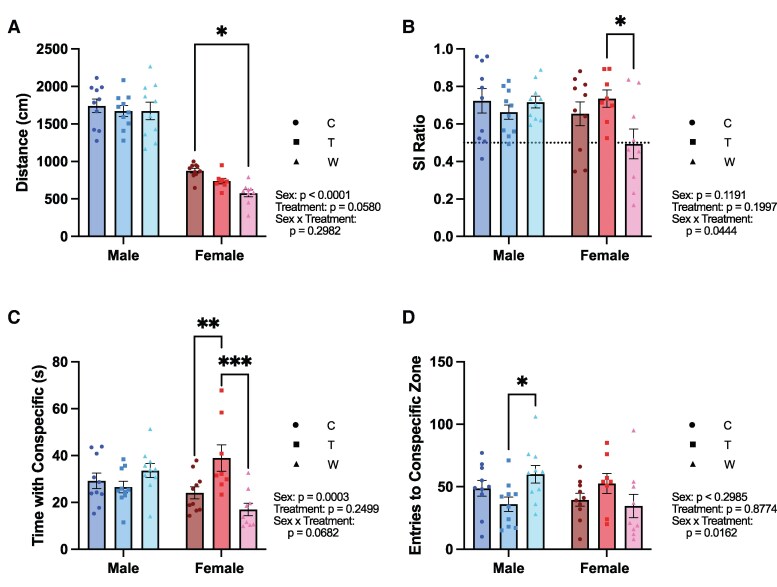
Social interaction test following 8 weeks of prednisolone treatment with or without withdrawal, or 8 weeks of vehicle treatment. A, Female mice in withdrawal explored the arena less than control females. B, Female mice undergoing withdrawal spent less time interacting with a novel conspecific compared to females receiving prednisolone treatment and males in withdrawal. C, Female mice treated with prednisolone spent more time with a novel conspecific than both control and withdrawal groups. D, Prednisolone treatment decreased the number of entries to the conspecific zone in male mice. Statistical results listed on the graphs are from 2-way ANOVA with sex and treatment as independent variables. Error bars represent SEM. *P < .05, **P < .005, ***P < .0005 in post hoc tests. Abbreviations: C, Control; T, Treatment; W, Withdrawal.

Having established that chronic prednisolone treatment recapitulates key effects of human glucocorticoid excess on metabolism and HPA axis suppression, we used several behavioral tests to measure the impact of this paradigm on motivated behavior, a symptom domain relevant to GWS. The first behavioral test performed was the social interaction test, which measures innate motivation to interact with a novel conspecific (Fig. 4). Preference for social interaction was measured using the social interaction ratio, where a ratio >0.5 indicates social preference [29].

A series of 2-way ANOVAs were used to examine the effect of sex and treatment condition on multiple behavioral measures including total distance traveled during the testing session, social interaction ratio, time spent with the novel conspecific, and entries to the conspecific zone. The results suggest a sex-dependent effect of glucocorticoid withdrawal on social behavior. Male mice traveled a significantly greater distance then the females (F[1, 51] = 240.2, *P* < .0001); however, there was no main effect of treatment condition (F[2, 51] = 3.013, *P* = .0580) or interaction between sex and treatment (F[2, 51] = 1.239, *P* = .2982) on this measure. There were no main effects of sex on the social interaction ratio (F[1, 51] = 2.512, *P* = .1191), time spent with the novel conspecific (F[1, 51] = 1.354, *P* = .2499), or entries to the conspecific zone (F[1, 51] = 1.103, *P* = .8774). There were also no main effects of treatment on the social interaction ratio, (F[2, 51] = 1.663, *P* = .1997), time spent with the novel conspecific (F[2, 51] = 2.832, *P* = .0682), or entries to the conspecific zone (F[2, 51] = 0.1311, *P* = .8774). There were, however, interactions between sex and treatment on all 3 measures, suggesting a difference in the effect of treatment on social interaction between the sexes (social interaction ratio, F[2, 51] = 3.313., *P* = .0444; time with novel conspecific, F[2, 51] = 9.548, *P* = .0003; entries to conspecific zone, F[2, 51] = 4.478, *P* = .0162).

For the SI ratio, post hoc comparisons revealed that the female withdrawal group had less preference for the novel conspecific than female treatment group (*P* = .0165). To determine whether the preference for social interaction exceeded the chance value, 1-sample *t* tests were conducted for each group separately by sex. These analyses revealed that all groups had a preference for the novel conspecific except the female withdrawal group (male control: *t*[9] = 3.418, *P* = .0076; male treatment: *t*[9] = 4.260, *P* = .0021; male withdrawal: *t*[9] = 6.923, *P* < .0001; female control: *t*[9] = 2.439, *P* = .0374; female treatment: *t*[7] = 5.136, *P* = .0013; female withdrawal: *t*[8] = 0.08023, *P* = .9380). These findings demonstrate that prednisolone withdrawal decreased social preference specifically in female mice.

The female treatment group spent significantly more time with the novel conspecific compared to female controls (*P* = .0088) and the female withdrawal group (*P* = .0001) suggesting that chronic prednisolone treatment increased motivation to explore a novel conspecific specifically in female mice. The female withdrawal group spent the least amount of time exploring the novel conspecific, consistent with the finding from the SI ratio that prednisolone withdrawal decreased social motivation in female mice. The male withdrawal group made more entries into the conspecific zone than males in the treatment group (*P* = .0399).

Collectively, these findings suggest that chronic prednisolone withdrawal differentially impacts social behavior in male and female mice. Female mice on prednisolone treatment showed increased interaction with a novel conspecific compared to the control group. During glucocorticoid withdrawal, this effect diminished falling below control levels. Females in withdrawal showed reduced social motivation, including less interaction with a novel conspecific and a lack of preference for a conspecific compared to an empty cup. These differences were not seen in males.

### Chronic Prednisolone Treatment Decreases Voluntary Wheel Running

**Figure 5.**
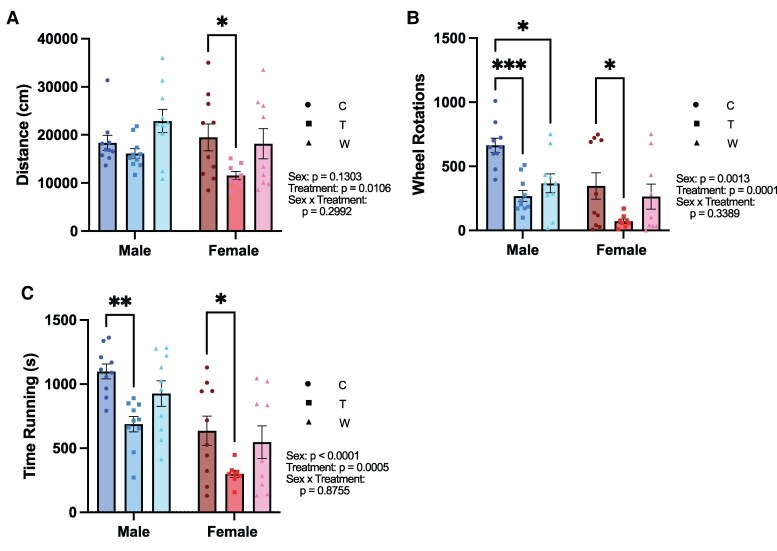
Prednisolone treatment decreases voluntary wheel running activity, partially reversed by withdrawal. A, Female mice treated with prednisolone explored the arena less than both control and withdrawal females. B, Prednisolone treatment reduced the number of wheel rotations in both male and female mice. C, Male and female mice receiving prednisolone treatment spent less time running on the wheel compared to control. Statistical results listed on the graphs are from 2-way ANOVA with sex and treatment as independent variables. Error bars represent SEM. *P < .05, **P < .005, ***P < .0005 in post hoc tests. Abbreviations: C, Control; T, Treatment; W, Withdrawal.

Voluntary wheel running was used as an additional measure of motivated behavior, as mice are innately motivated to run (Fig. 5). Two-way ANOVAs were conducted to assess the effects of sex and treatment condition on total distance traveled, number of wheel rotations, and time spent running on the wheel.

Prednisolone treatment significantly reduced voluntary wheel running in both sexes. Males generally exhibited higher activity levels than females, with significant main effects of sex on number of wheel rotations (F[1, 51] = 11.66, *P* = .001) and time spent running (F[1, 31] = 30.92, *P* < .0001), though there was no significant effect of sex on total distance traveled (F[1, 51] = 2.365, *P* = .1303). There was a main effect of treatment on distance traveled (F[2, 51] = 4.979, *P* = .0106), wheel rotations (F[2, 51] = 10.56, *P* = .0001), and time spent running (F[2, 51] = 8.741, *P* = .0005). There were no interactions between sex and treatment on any measure, suggesting that prednisolone affected voluntary wheel running behavior similarly in both sexes: distance (F[2, 51] = 1.236, *P* = .2992); wheel rotations (F[2, 51] = 1.105, *P* = .3389); time spent running (F[2, 51] = 0.1333, *P* = .8755).

Prednisolone-treated mice consistently exhibited the lowest levels of wheel running behavior across all measures and both sexes. In males, prednisolone treatment significantly decreased wheel rotations (*P* = .0007), and time spent running (*P* = .0047) relative to controls. Although this effect appeared to partially reverse during withdrawal, withdrawal males still showed significantly fewer wheel rotations compared to control males (*P* = .0126). A similar pattern was seen in the females, where treatment significantly reduced distance traveled (*P* = .0394), wheel rotations (*P* = .0349), and time spent running (*P* = .0354) relative to the control group, with a partial reversal of this effect during withdrawal. In summary, chronic prednisolone treatment decreased voluntary wheel running behavior. This decrease was evident in both sexes and appears to be partially reversed by day 4 of withdrawal.

### Mechanical Pain Sensitivity Increases During Prednisolone Withdrawal

**Figure 6.**
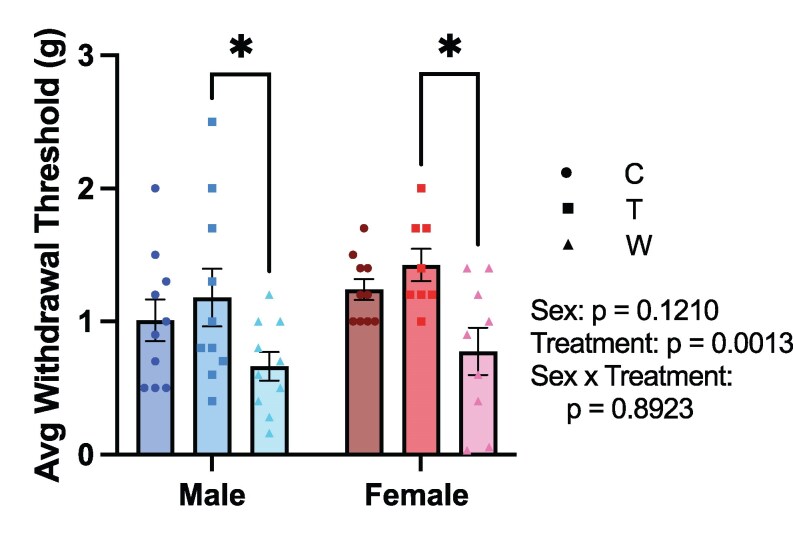
Mechanical pain sensitivity was increased during prednisolone withdrawal. Statistical results listed on the graphs are from 2-way ANOVA with sex and treatment as independent variables. Error bars represent SEM. *P < .05 in post hoc tests. Abbreviations: C, Control; T, Treatment; W, Withdrawal.

Pain, including diffuse myalgias and arthralgias, is one of the most prominent symptoms of the GWS, independent of underlying disease activity [7, 8]. To assess mechanical pain sensitivity in mice undergoing the current withdrawal paradigm, we used the von Frey ascending stimulus method [30, 31] (Fig. 6). A significant main effect of treatment (F[2, 54] = 7.476, *P* = .0014) on mechanical pain sensitivity in the von Frey was observed; however, there was no main effect of sex (F[1, 51] = 2.487, *P* = .1210) or a significant interaction between sex and treatment (F[2, 51] = 0.1142, *P* = .8923) on average paw withdrawal thresholds. Post hoc testing revealed that mice in the withdrawal groups had significantly lower paw withdrawal thresholds compared to the treatment groups regardless of sex (males: *P* = .0437; females: *P* = .0165), indicating increased mechanical pain sensitivity. These results demonstrate that glucocorticoid withdrawal significantly increased pain sensitivity in mice, recapitulating a core symptom of GWS in humans.

### Prednisolone Treatment and Withdrawal Has No Effect on Affective Behavior

**Figure 7.**
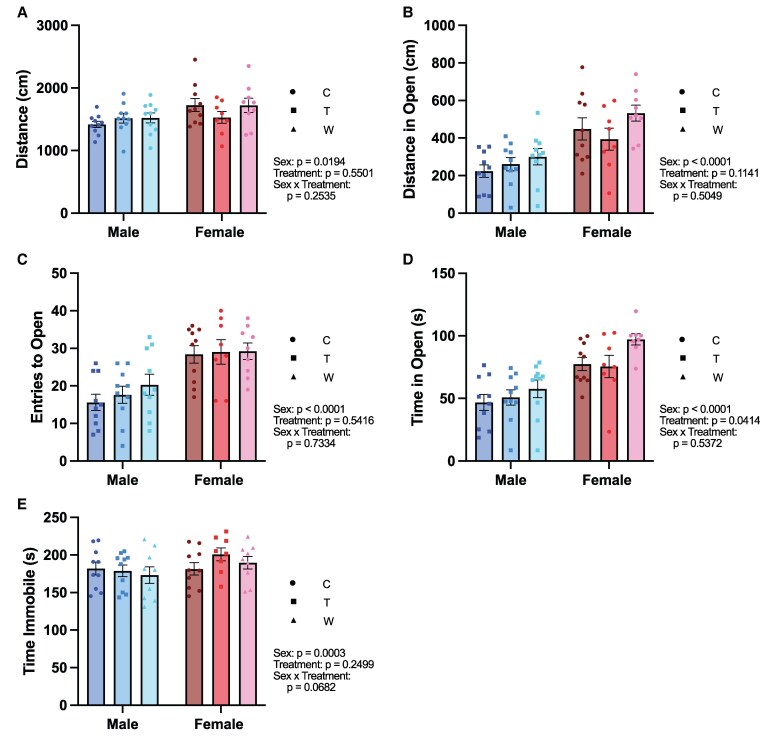
Affective behavior was not changed following prednisolone treatment and withdrawal. In the elevated zero maze, there were no significant effects of glucocorticoid treatment or withdrawal on: A, total distance traveled, B, distance traveled in the open zone, C, number of entries to the open. D, There was a significant effect of treatment on time spent in the open. E, There were no differences in time spent immobile during the tail suspension test. Statistical results listed on the graphs are from 2-way ANOVA with sex and treatment as independent variables. Error bars represent SEM. *P < .05 in post hoc tests. Abbreviations: C, Control; T, Treatment; W, Withdrawal.

Chronic glucocorticoid excess is associated with increased risk for depression and anxiety in humans [42-44]. Previous research has shown that chronic corticosterone administration induces negative affective behaviors in rodents, including anxiety- and depressive-like phenotypes [45-52]. Psychiatric symptoms have also been reported during glucocorticoid withdrawal; however, it remains unclear if these symptoms reflect true affective disturbances or are instead linked to fundamental changes in energy and motivation [7, 8]. To assess affective behavior following chronic prednisolone treatment and withdrawal, we used the elevated zero maze and tail suspension test (Fig. 7).

In the elevated zero maze, males explored the arena less than females, seen as a significant main effect of sex in all measures: total distance traveled (F[1, 51] = 5.830, *P* = .0194), distance traveled in the open arms (F[1, 51] = 27.51, *P* < .0001), entries to the open arms (F[1, 51] = 28.85, *P* < .0001), and time spent in the open arms (F[1, 50] = 35.20, *P* < .0001). Additionally, there was also an effect of treatment on time spent in the open arms (F[2, 50] = 3.395, *P* = .0414); however, post hoc comparisons did not reveal significant differences between the groups. In the tail suspension test, there was no significant effect of sex (F[1, 50] = 3.085, *P* = .0852), treatment (F[1, 50] = 0.5764, *P* = .5656), or an interaction between sex and treatment (F[2, 50] = 0.9049, *P* = .4111) on time spent immobile, suggesting no clear treatment- or sex-related differences in depressive-like behavior.

Together, these findings suggest a minimal effect of chronic prednisolone treatment and withdrawal on affective behavior in this paradigm. Thus, the behavioral manifestations observed in other tests, such as the social interaction test and von Frey, are unlikely to be explained by underlying affective changes.

### Region-Specific Changes in GR Immunoreactivity During Prednisolone Withdrawal

**Figure 8.**
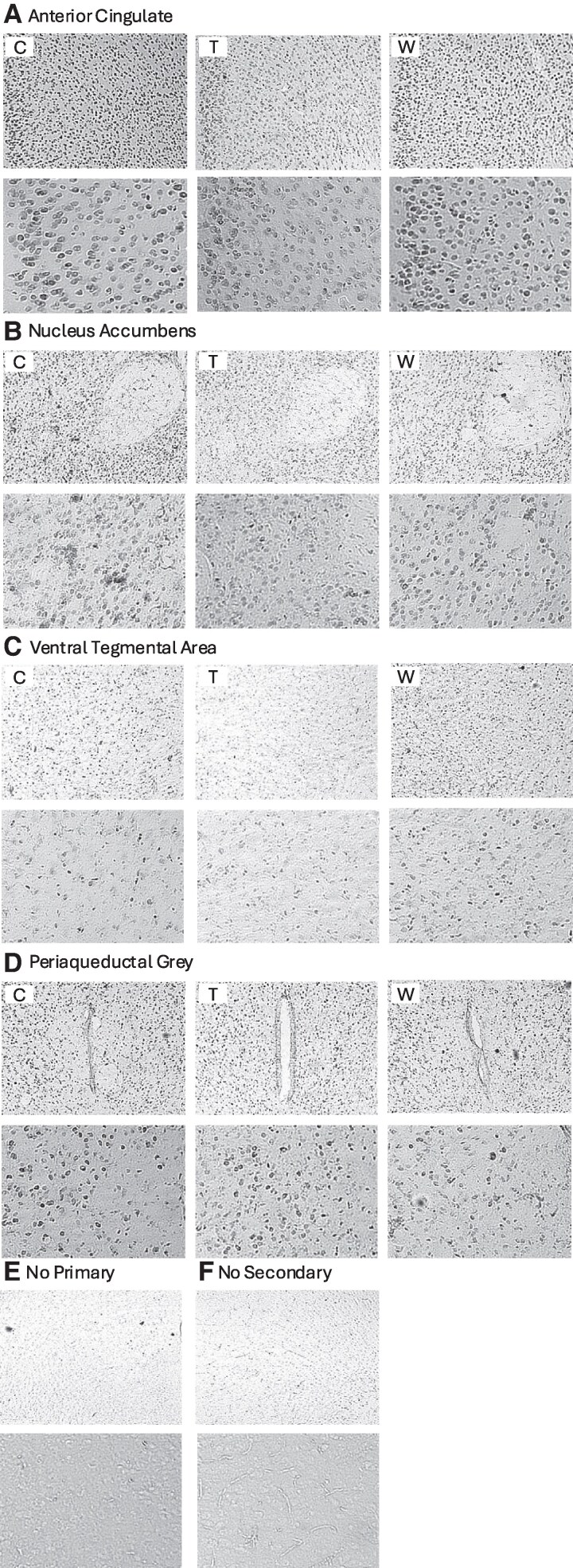
Representative images showing GR immunolabeling in each brain region including the A, anterior cingulate cortex, B, nucleus accumbens, C, ventral tegmental area, and D, periaqueductal gray. Additional sections representing € no primary and (F) no secondary controls. In each panel, top and bottom images were taken using 4× and 10× objectives, respectively.

**Figure 9.**
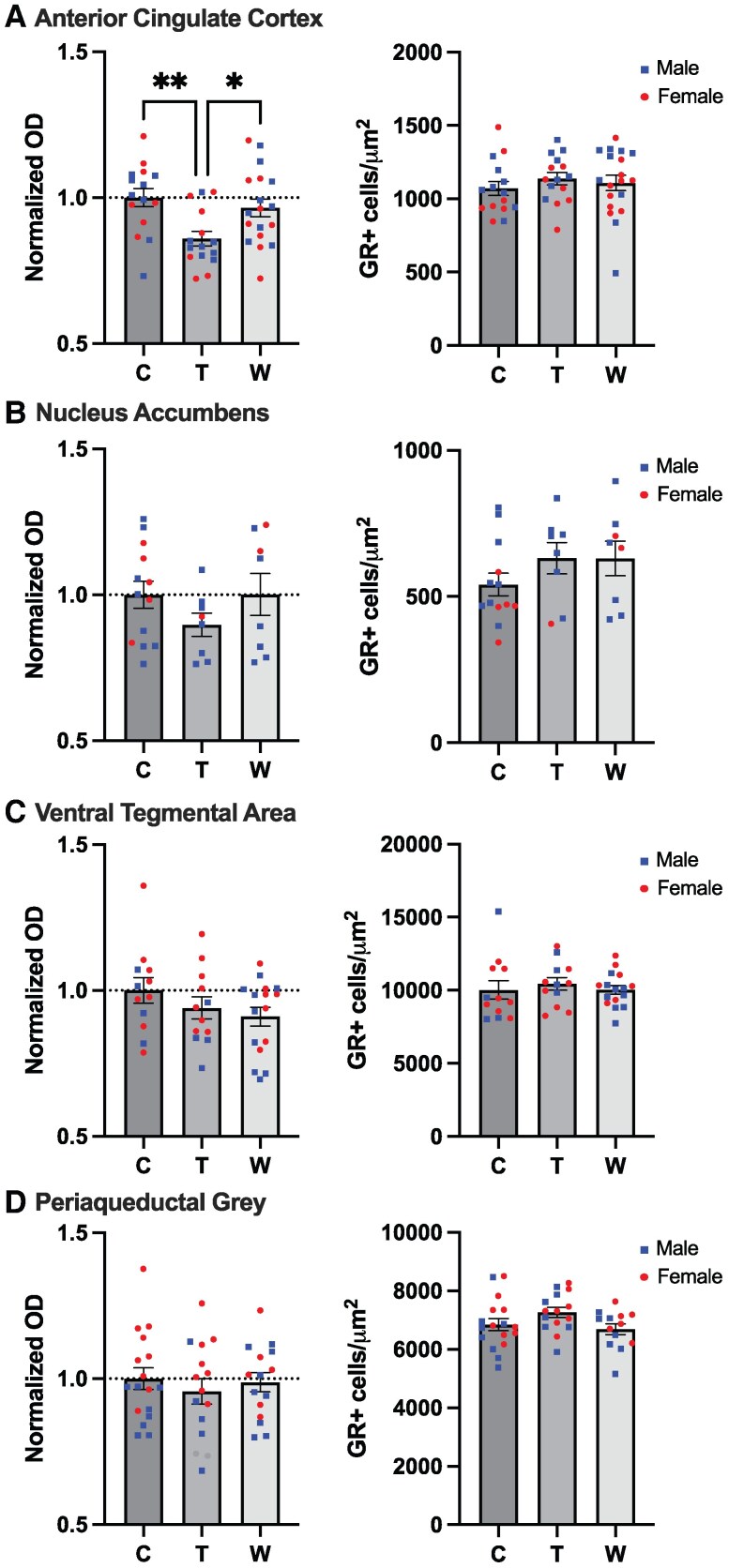
Changes in glucocorticoid receptor immunoreactivity following prednisolone treatment and withdrawal. A, There was a significant decrease in GR+ cell expression in the ACC during prednisolone treatment with no change in optical density. There were no significant effects of prednisolone treatment or withdrawal on GR+ cell expression or optical density in the B, nucleus accumbens, C, ventral tegmental area, or D, periaqueductal gray. Error bars represent SEM. *P < .05 in post hoc tests.

Having developed a mouse model that captures key features of GWS, we next sought to use this model to test a potential underlying mechanism. We measured GR expression using GR immunolabeling and quantified the optical density of GR immunoreactivity (GR-ir) and the number of GR-expressing cells (cell counts per total area) in brain regions previously implicated in pain, motivation, or both, including the nucleus accumbens (NAc), anterior cingulate cortex (ACC), ventral tegmental area (VTA), and periaqueductal gray (PAG) (Figs. 8 and 9). For GR-ir optical density, a significant main effect of treatment was observed in the ACC (F[2, 45] = 5.938, *P* = .0051). Post hoc comparisons revealed a significant decrease in GR immunoreactivity in the treatment group compared to both control (*P* = .0054) and withdrawal (*P* = .0341) groups. No significant effects of treatment were observed in the NAc (F[2, 25] = 1.138, *P* = .3360), VTA (F[2, 37] = 1.508, *P* = .2346), or PAG (F[2, 44] = 0.3557, *P* = .7027). There were no significant effects of treatment on GR + cell counts in any of the regions examined: ACC (F[2, 46] = 0.4468, *P* = .6424); NAc (F[2, 26] = 1.268, *P* = .2982); VTA (F[2, 37] = 0.2665, *P* = .7675); and PAG (F[2, 42] = 2.306, *P* = .1121). These results suggest that the number of GR-expressing cells remains unchanged following prednisolone treatment or withdrawal. In summary, these data suggest a brain region–specific effect of prednisolone treatment to decrease GR-ir but not the total number of GR-expressing cells specifically in the ACC in male and female mice, an effect which reverses within 1 week of withdrawal.

## Discussion

Glucocorticoid withdrawal syndrome is one of the reasons why millions of patients around the world continue to take long-term glucocorticoids despite their significant health and economic cost and the increasing availability of steroid-sparing treatments for inflammatory and autoimmune diseases. There are currently no treatments available for the GWS, and we do not know its mechanisms. Here, we present the first animal model developed to study the mechanisms of the GWS. We found that mice exhibit behavioral phenotypes during glucocorticoid withdrawal that recapitulate key aspects of the human syndrome, including increased pain sensitivity and decreased social motivation—the latter in females only. These findings suggest that this novel animal model can be used to investigate the mechanisms underlying GWS and to evaluate potential therapeutic interventions.

The effects of chronic glucocorticoid exposure in mice vary depending on the specific glucocorticoid used and duration of treatment [36, 53-56]. Chronic corticosterone treatment usually leads to weight gain in mice; however, with other steroids, weight loss has also been reported. Universally, chronic glucocorticoid treatment results in an increased fat-to-lean body mass ratio. In our study, chronic prednisolone treatment initially caused weight loss, which was subsequently regained. This was accompanied by the expected metabolic consequence of insulin resistance.

While we observed a significant reduction in circulating corticosterone levels in mice undergoing chronic prednisolone treatment, consistent with expected HPA axis suppression, we also found a sex difference in the degree of suppression. After 4 weeks of treatment, females exhibited more complete suppression of the diurnal corticosterone rise than males, despite receiving the same concentration of prednisolone in the drinking water. As expected, based on known sex differences, females had higher corticosterone levels than males. There were several control female mice with very high afternoon corticosterone levels, which could indicate an HPA axis response to the blood sampling procedure; of note, this was not seen in any prednisolone-treated females, suggesting that during treatment the prednisolone suppressed both procedural stress-induced and diurnal corticosterone production. On the other hand, at the time of perfusion, treated females did not show statistically significant HPA axis suppression despite ongoing prednisolone treatment, potentially indicating greater suppression of the HPA axis response to the perfusion procedure in treatment males relative to females. Previous literature has demonstrated a positive correlation between body size and fluid intake, suggesting that prednisolone exposure may have varied between the sexes and over time due to metabolic and intake differences [57]. Unfortunately, we were not able to measure circulating levels of prednisolone in these mice. To our knowledge, sex differences in HPA axis suppression by exogenous glucocorticoids have not been systematically studied. Such differences could influence the risk of prolonged adrenal insufficiency and the GWS phenotype.

Although our rationale for continuing low-dose prednisolone during the withdrawal phase was to avoid adrenal insufficiency, it remains possible that we only prevented peripheral, but not central, hypocortisolemia. Prior studies have suggested that low concentrations of the synthetic glucocorticoids prednisolone and dexamethasone can be actively excluded from the blood brain barrier [58-62]. As a result, central hypocortisolemia may occur even when circulating levels remain within physiological range. Our perfusion corticosterone data suggest that by 9 days into withdrawal, there is at least a partial recovery of HPA axis function in the withdrawal group, which would at that point prevent complete central hypocortisolemia, though we cannot say from this data when during withdrawal this recovery begins. This data does suggest that, like in humans, the behavioral manifestations of GWS may be independent of HPA axis recovery in mice. To that end, future studies measuring plasma corticosterone at multiple time points during withdrawal, along with assessment of brain glucocorticoid exposure or receptor activity, will be important.

Our behavioral characterization encompassed tests of motivated behavior and pain sensitivity, as decreased behavioral motivation and increased pain are 2 of the most prominent symptoms observed in GWS. We found evidence of decreased social motivation during glucocorticoid withdrawal in female mice, measured as decreased interaction with a novel conspecific, which was not seen in males. Sex differences in the rewarding properties of social interaction in mice have previously been observed. Previous literature using a social conditioned place preference test found that female mice showed less social seeking and social reward than male mice [63]. A lower baseline motivation for social contact could explain the increased sensitivity to glucocorticoid withdrawal in females observed in the social interaction test. The sex differences reported here may also stem from underlying differences in the neural circuitry governing social motivation, from differential effects of chronic glucocorticoid treatment and withdrawal, or from sex-based variation in prednisolone exposure within our model. While sex differences in the GWS have not previously been systematically studied, its prevalence is expected to be higher in females, in line with the female predominance of both endogenous Cushing disease and many autoimmune diseases.

We also saw decreased wheel running during prednisolone treatment, which partially reversed during withdrawal. Consistent with our findings, decreased locomotor activity in the home cage and on a running wheel has previously been reported following week-long corticosterone treatment via drinking water [64]. These results contrast with our findings on social motivation, indicating that glucocorticoid withdrawal does not uniformly decrease motivated behavior in mice; rather, such effects appear to be domain- and sex-specific. The mesolimbic dopamine system is thought to govern motivation for multiple naturally rewarding stimuli [65]. Previous literature has demonstrated the importance of the VTA and its dopaminergic projections to the nucleus accumbens in driving both social behavior and locomotor activity, which we assessed in our study [66-69]. Our findings suggest that the symptoms of GWS may not be due to broad changes in this central motivational circuitry, as such changes would likely affect both wheel running and social interaction [69, 70]. However, glucocorticoid withdrawal likely affects multiple neural circuits, including potentially those that control locomotor activity, which can result in a more nuanced behavioral phenotype. It is also possible that the effects of glucocorticoid withdrawal on motivated behavior are species-specific.

The most prominent phenotype observed during glucocorticoid withdrawal in our model was in the domain of pain, an accurate reverse translation from the human GWS. Myalgias and arthralgias are the most common symptoms of the GWS, affecting approximately 50% of patients during withdrawal [71]. This increase in pain sensitivity is often generalized and lacks a clear origin of nociceptive input. Central sensitization may account for this phenomenon, potentially arising from enhanced neural signaling, reduced inhibition, or both [72-74]. Our findings of increased mechanical pain sensitivity in mice during glucocorticoid withdrawal supports the idea that sensory processing within the central nervous system may be altered in this context, as no peripheral injury has occurred to elicit this increased mechanical sensitivity. Future work using this model will help elucidate the mechanisms underlying this increased pain sensitivity during glucocorticoid withdrawal, which contributes significantly to physical impairment and reduced quality of life in patients experiencing GWS.

Finally, using the elevated zero maze and the tail suspension test, we found no evidence that chronic prednisolone treatment or withdrawal altered innate affective behavior. Chronic corticosterone exposure is often used to induce a depressive-like phenotype in mice, which could make our results surprising [45, 46, 49]; however, differences between these glucocorticoids, including the longer half-life of prednisolone, could account for discrepancies with the existing literature. Additionally, most prior studies assessed affective behavior at earlier time points (commonly after 3-4 weeks of corticosterone administration). Nonetheless, our results support the interpretation that the observed changes in social interaction and mechanical pain sensitivity during glucocorticoid withdrawal were independent of a generalized increase in negative affective behavior.

The GWS is likely a GR-dependent phenomenon. Brain regions exhibiting adaptive decreases in GR expression during chronic prednisolone treatment would be particularly vulnerable to a sudden decrease in glucocorticoid availability during withdrawal [24, 25, 75-77]. To see if this adaptive GR downregulation occurs during chronic prednisolone treatment in brain regions implicated in the studied behaviors, we used GR immunolabeling on brain sections. Western blotting could also be used for protein quantification, but it provides limited anatomic resolution or, in the case of punched tissue from neural subregions, samples only a small portion of the target brain region. We found that there were not widespread changes to GR expression in our model, but there was a significant decrease in the optical density of GR immunoreactivity during prednisolone treatment specifically in the anterior cingulate cortex, without any change in the number of GR+ cells. While this technique does not distinguish between cytoplasmic and nuclear GR, nonetheless, the findings suggest an overall decrease per cell in the amount of GR protein.

The ACC plays a critical role in social behavior [78, 79]. It is also an important part of pain circuitry, where it is particularly involved in the affective components of pain [80-83]. We suggest that GR downregulation in the ACC during prednisolone treatment contributes to the behavioral manifestations of GWS observed here in social interaction and mechanical pain sensitivity, a hypothesis that will need to be tested. One limitation to this conclusion is that this prednisolone-induced decrease in GR expression had apparently normalized after 1 week of glucocorticoid withdrawal, and we do not know the timing of this normalization relative to the behavioral testing. It is also possible that the changes in GR expression and its recovery are cell-type specific, as the functional heterogeneity of neurons in the ACC has previously been established. For example, activation of excitatory ACC neurons has been shown to lower the mechanical pain threshold, whereas activation of inhibitory interneurons in the ACC during a chronic pain state could alleviate pain [84-86]. These findings highlight the importance of examining region- and cell-type-specific molecular and functional changes to better understand the neural circuitry underlying the GWS. The functional importance of GR signaling in the ACC for the GWS phenotype will be an important direction for future study.

## Conclusion

The significant conservation of glucocorticoid biology between mice and humans led us to develop an animal model of the GWS to investigate its underlying mechanisms. The model presented here offers a faithful reverse translation of a key GWS symptom, increased pain sensitivity in both sexes and decreased motivated behavior in females. This suggests that critical aspects of GWS biology are conserved across species. We further demonstrated the utility of this model for testing mechanistic hypotheses, identifying decreased GR expression in the ACC during treatment as a potential mechanism. This work lays the foundation for a biological understanding of the GWS and the future development of targeted treatments.

## Data Availability

Some or all datasets generated during and/or analyzed during the current study are not publicly available but are available from the corresponding author on reasonable request.

## Abbreviations

ACC: anterior cingulate cortex
GR: glucocorticoid receptor
GR-ir: glucocorticoid receptor immunoreactivity
GWS: glucocorticoid withdrawal syndrome
HPA: hypothalamic-pituitary-adrenal
NAc: nucleus accumbens
PAG: periaqueductal gray
PBS: phosphate-buffered saline
TBS: Tris-buffered saline
VTA: ventral tegmental area
